# Connecting nutrition composition measures to biomedical research

**DOI:** 10.1186/s13104-018-3997-y

**Published:** 2018-12-12

**Authors:** Jeremy J. Jay, Alexa Sanders, Robert W. Reid, Cory R. Brouwer

**Affiliations:** Department of Bioinformatics and Genomics, College of Computing and Informatics, University of North Carolina at Charlotte, Charlotte, NC USA

**Keywords:** Nutrition, Nutrient, Diet, Food crops, Agriculture, Nutrition informatics

## Abstract

**Objectives:**

Biomedical research is gaining ground on human disease through many types of “omics”, which is leading to increasingly effective treatments and broad applications for precision medicine. The majority of disease treatments still revolve around drugs and biologics. Although food is consumed in much higher quantities, we understand very little about how the human body metabolizes and uses the full range of nutrients, or how these processes affect human health and disease risk. Nutrient composition databases are used by dietitians to describe common consumer food products, but these fail to identify chemicals with the same nomenclature as metabolic pathways in basic life sciences research and with far less precision. Consumer-oriented nutrient compositions often describe generic substances (e.g. Sugars) while scientific reporting is often much more specific (e.g. Dextrose, Fructose, etc.). Integrating these two fields of research presents a difficult challenge for novel applications of precision nutrition.

**Data description:**

This data set provides a manually curated collection of nutrient identifiers from the USDA’s Nutrition Data Bases and maps them to PubChem (a resource for cheminformatics and drug discovery research), biomedical literature records in PubMed using Medical Subject Headings, biological pathways using the Chemical Entities of Biological Interest ontology.

## Objective

Biomedical research is gaining ground on human disease through many types of ‘omics, leading to increasingly effective treatments and broad applications for precision medicine. However, the majority of disease treatments still revolve around drugs and biologics. Food is consumed in much higher quantities and yet we understand very little of the specifics about how the human body metabolizes and uses nutrients, and how these processes affect human health and disease. Understanding this better is one of the primary goals of the Plant Pathways Elucidation Project (P2EP) [1].

The P2EP Knowledgebase aims to collect and integrate data from food crops, nutrients, biochemical pathways and reactions, and human health and disease. This is a large undertaking that will require years of effort and data collection starting with genome assembly for many crop plants, and gene mapping and annotation to determine pathway representation. In order to jumpstart the informatics component of this project, we have integrated existing consumer-oriented nutrient composition databases with life sciences and biomedical resources.

Nutrient composition databases [2, 3] often describe food nutrients at a high level of generality (e.g. “Sugars”) while scientific reporting is often much more specific (e.g. “Dextrose”, “Fructose”, etc.). In addition, consumer reporting lists broad classifications that are not immediately useful in a scientific context (e.g. the broad spectrum of chemicals denoted by “trans fats”). Translating between these two worlds is a laborious process, but immensely valuable as more work is done towards precision nutrition.

Nutrition intersects various biomedical domains: basic science research, biomedical literature, and drug discovery and cheminformatics. There are many biological pathways resources such as the Gene Ontology [4] and Reactome Knowledgebase [5], which can be associated with the Chemical Entities of Biological Interest (ChEBI) ontology [6]. The Medical Subject Headings (MeSH) are a comprehensive terminology for the biomedical literature applied to the over 28 million articles in PubMed [7]. PubChem is a large resource providing further links to various cheminformatics and drug discovery data sets, tools, and applications [8]. Including these three public resources allows our mappings to be used with a wide variety of scientific resources.

## Data description

Our baseline sample of nutrient identifiers and names were extracted from multiple USDA Nutrient Databases. The primary resource was the Standard Reference 28 [2], which was supplemented with the contents of the Special Interest Databases on Flavonoids [3]. Together these records contained 188 unique nutrient identifiers related to 7793 foods in the standard reference database. Table 1 contains a listing of the resources produced.

**Table 1:** Overview of data files/data sets

Label	Name of data set	File types (file extension)	Data repository and identifier (DOI)
Data set 1	MeSH to USDA NDB mappings	.csv	10.6084/m9.figshare.7033190 [11]
Data set 2	ChEBI to USDA NDB mappings	.csv	10.6084/m9.figshare.7033208 [12]
Data set 3	PubChem CID to USDA NDB mappings	.csv	10.6084/m9.figshare.7033217 [13]

The list of Nutrient Identifiers and Names were given to two student interns of the Plant Pathways Elucidation Project (P2EP) [1]. The students were instructed to independently search the ChEBI web portal [6] and record the appropriate matching identifiers, making sure to select the appropriate biologically relevant enantiomers and avoid erroneous ions. As expected for some fatty acids, naming conventions varied between USDA documentation and the ChEBI nomenclature, for which manual research and annotation was performed to confirm. Student results were then cross-compared to verify and investigate differences, then confirmed by a domain scientist. After an initial technical validation against pathway databases, we modified the ChEBI annotations to use more general ancestor classifications for many nutrients, which allowed for variation in ambiguity, bioavailability, and in turn greater access to other linked resources. Approximately 22% of NDB Nutrients have no ChEBI equivalent.

The prior techniques were then repeated for assignment to Medical Subject Headings (MeSH) descriptors [7]. When multiple terms were available, we preferred the term with higher occurrence in PubMed metadata records (e.g. elemental “Phosphorus” has over 10 × more annotations than “Phosphorus, Dietary”). These counts were accessed from the MeSH browser at the web portal using the “Related Information” sidebar link to PubMed. Approximately 57% of NDB Nutrients have no equivalent MeSH identifier, as expected since MeSH is used for generic document annotation and not pathway mapping.

Finally, links to the PubChem Compound database [8] were collected using the prior mappings to MeSH and ChEBI as starting points. When multiple PubChem Compound IDs were available, the CID with the most appropriate IUPAC name was selected. PubChem records were also cross-referenced with AOAC International references as specified in the NDB documentation and Chemical Abstracts Service (CAS) identifiers [9]. Approximately 33% of NDB Nutrients have no PubChem annotations.

Interestingly, none of the resources annotated contains a complete matching to all of the USDA Nutrients listed. Twenty-nine of the NDB nutrients have no annotations to ChEBI, MeSH, or PubChem. While in some cases this can be attributed to the generic consumer-oriented nutrient classifications (8/29 NDB identifiers are terms such as Ash, Energy, Sugars, etc.), the remaining instances may be classifications that warrant further consideration into the scientific databases. Now that this resource exists, we can do further research into the properties and connections of these nutrients and find more information about the effects of both known and uncharacterized phytochemicals on human health.

## Limitations

The high specificity of scientific terminology does not map cohesively to the current level of ambiguity in the Nutrition Composition databases. While it is not immediately obvious that adding new generic terminology to existing resources like ChEBI or MeSH is beneficial, adding more comprehensive lists of chemical variations with similar bioavailability would be desirable for increasing utility and accessibility of more diverse data sets.

## Abbreviations

USDA: United States Department of Agriculture
NDB: Nutrient Database (aka USDA National Nutrient Database for Standard Reference)
MeSH: Medical Subject Headings
ChEBI: Chemical Entities of Biological Interest
CAS: Chemical Abstracts Service
P2EP: Plant Pathways Elucidation Project
IUPAC: International Union of Pure and Applied Chemistry
